# Effects of probiotic supplementation on lipid profile of women with rheumatoid arthritis: A randomized placebo-controlled clinical trial

**DOI:** 10.15171/hpp.2017.17

**Published:** 2017-03-05

**Authors:** Elnaz Vaghef-Mehrabany, Leila Vaghef-Mehrabany, Mohammad Asghari-Jafarabadi, Aziz Homayouni-Rad, Karim Issazadeh, Beitullah Alipour

**Affiliations:** ^1^Department of Nutrition, Biochemistry and Diet Therapy, Tabriz University of Medical Sciences, Tabriz, Iran; ^2^Department of Clinical Nutrition, Tehran University of Medical Sciences, Tehran, Iran; ^3^Road Traffic Injury Research Center, Tabriz University of Medical Sciences, Tabriz, Iran; ^4^Department of Food Science and Technology, Tabriz University of Medical Sciences, Tabriz, Iran; ^5^Faculty of Nutrition and Food Sciences, Tabriz University of Medical Sciences, Tabriz, Iran; ^6^Department of Community Nutrition, Tabriz University of Medical Sciences, Tabriz, Iran

**Keywords:** Cholesterol, Lactobacillus casei, Probiotics, Rheumatoid arthritis, Serum lipoproteins

## Abstract

**Background:** Probiotics are live beneficial microorganisms which may exert hypolipidemic effects through many mechanisms. Lipid profile disturbances are frequently reported in rheumatoid arthritis (RA) patients. The objective of this study was to evaluate the effects of Lactobacillus casei on serum lipids of RA women.

**Methods:** In the present parallel randomized double-blind placebo-controlled clinical trial, 60 RA patients were recruited and divided into 2 groups. They received either a daily capsule containing 10^8^ CFU of L. casei 01, or identical capsules containing maltodextrin, for 8 weeks. Anthropometric parameters, dietary intake and physical activity were assessed at 2 ends of the study. Serum levels of total cholesterol (TC), high-density lipoprotein-cholesterol (HDL-C), low-density lipoprotein-cholesterol (LDL-C) and triglyceride (TG) were measured. Independent-samples t test and analysis of covariance (ANCOVA) test, and paired t test were used to test between- and within-group differences, respectively.

**Results:** There were no significant between- or within-group differences for demographic and anthropometric parameters, physical activity and dietary intakes, throughout the study. No statistically significant within-group changes were observed for serum lipids in either group; between-group differences were also insignificant by the end of study period (TC: -0.18 [-0.65, 0.29], P = 0.801, HDL-C: -1.66 [-19.28, 15.59], P = 0.663, LDL-C: -2.73 [-19.17, 13.73], P = 0.666, TG: 0.12 [-19.76, 20.00], P = 0.900).

**Conclusion:** Lactobacillus casei 01 could not improve serum lipids in RA patients. Further studies using probiotic foods and different probiotic strains are suggested.

## Introduction

Rheumatoid arthritis (RA) is a common systemic inflammatory disease characterized by severe pain in joints and functional disability; it is of unknown etiology and affects 0.5%-1.0% of adults.^1,2^ Cardiovascular diseases (CVD) are prevalent among RA patients and account for approximately half of the deaths in RA.^3^ Lipid profile abnormalities particularly lower levels of high-density lipoprotein-cholesterol (HDL-C) have been frequently reported in RA patients.^4^ Moreover, RA patients have greater limitations in increasing their physical activity, due to their extremely painful joints, which increases their risk of developing CVD.^4-8^ Timely screening and relevant interventions to keep serum lipids at desirable levels is critical.

Probiotics are “live microorganisms which, when administered in adequate amounts, confer a health benefit on the host”^9^; these benefits are strain-specific.^10^ Clinical trials investigating beneficial effects of probiotics on lipid profile in both healthy and hyperlipidemic participants have come up with promising results.^11-14^ A systematic review by Guo et al revealed that probiotics can reduce plasma total cholesterol (TC) and low-density lipoprotein-cholesterol (HDL-C) in subjects with normal, borderline high and high cholesterol levels.^15^ Strains of the species *Lactobacillus casei* have shown hypolipidemic effects in some animal studies. Feeding *L. casei* TMC 0409 fermented milk suppressed cholesterol elevation in rats under high cholesterol diet; HDL-C was significantly increased in the probiotic group and serum triglyceride (TG) was insignificantly decreased.^16^ In hamsters, skim milk fermented with *L. casei* Shirota decreased plasma TG in both cholesterol-free and cholesterol-enriched diets.^17^ Also, feeding lyophilized *L. casei* subsp. *casei* for 90 days, reduced cholesterol by 15%-25% in rats, compared to a skim milk group.^18^

RA is a prevalent disorder and the disease, its subsequent complications including CVD, and the side effects of the medications used for its treatment impose great medical and financial burden on the governments; thus it can be regarded as a community health problem demanding greater attention. We hypothesized that probiotics with beneficial effects on lipid profile may improve lipids in RA subjects. To the best of our knowledge, the effect of probiotic supplementation on plasma lipids of RA patients has not been studied thus far. The objective of the present clinical trial was to evaluate the effects of *L. casei* 01 supplementation on serum TG, TC, LDL-C and HDL-C in RA women. This study is a secondary analysis from a previously published study.^19^

## Materials and Methods

### Study design

This was a randomized double-blind, parallel group, placebo-controlled trial. No changes were applied in the study design or trial outcomes after the commencement of the study. Details of the study procedures, subject recruitment and exclusion criteria were presented previously.^19^

### Study participants

Those eligible for randomization met the following criteria: having established RA for more than one year, having inactive to moderate level of the disease, being 20-80 years old, having body mass index (BMI) lower than 40 and following stable medication for at least the preceding three months. Patients who were pregnant or lactating, under hormone therapy, suffering from metabolic or gastrointestinal diseases, taking antioxidant, vitamin, fiber or omega-3 supplements three weeks prior to the interventions, exposed to cigarette smoke, using antibiotics or other probiotic products and following a weight reduction diet were excluded from the study. With a confidence level of 95% and power of 80% and based on the mean (SD) results for HDL-C in the study by Simons et al, and assuming a 30% of probable withdrawal or drop out of patients during the study or analyses, sample size for the present clinical trial was calculated to be 22 in each group.^20^ Thus, the sample size that had been calculated for the primary study (n = 30),^19^ was adequate for the present trial. Thirty eligible subjects recruited from rheumatology clinic of Sina hospital and Sheykholrayis Polyclinic (Tabriz, Iran), were randomized in each group using a computer generated blocked randomization list stratified by 2 factors, menopausal status (premenopausal or postmenopausal) and BMI range (<30 vs. >30 kg.m^-2^); randomization was performed by the technician who measured anthropometric measures of the participants. The patients and those who assessed the outcomes were blind to the intervention assignments. The bottles containing either probiotic or placebo capsules were handed over to the patients, after their first visit to the clinic, baseline assessments and randomization. The patients were asked to adhere to their baseline medications, for the whole 8 weeks and report any changes in their drugs in terms of both type and dosage; this was necessary to control for the confounding effect of the treatments that the subjects received for RA or other co-morbidities.

### IPAQ, STAI-Y, dietary and anthropometric assessments

At baseline, a demographic questionnaire, the short form of International Physical Activity Questionnaire (IPAQ),^21^ Spielberger State-Trait Anxiety Inventory Form Y (STAI-Y),^22,23^ a 24-hour dietary recall questionnaire and 3 food record questionnaires were completed for the participants. Usual dietary intakes of the patients were estimated based on mean scores for calorie-nutrients obtained from the dietary questionnaires. Weight and height were also measured by Seca scale (Seca, Germany; with the precision of 500 g) and a tape measure (to the nearest 0.1 cm), respectively. The same assessments were performed after the 8 weeks of study duration.

### Study intervention

Probiotic capsules contained a minimum of 10^8^ colony forming unit (CFU) of *L. casei* 01 (Chr. Hansen; Horsholm, Denmark) and maltodextrin (Shandong; Shandong, China) while the placebo capsules which were exactly identical to probiotic ones consisted of only maltodextrin. The bacterial count of probiotic capsules at baseline, in the middle and at the end of the intervention period, confirmed the acceptable number of probiotics in each capsule during the study course. To assess compliance with the study protocol, the remaining capsules in the participants’ bottles were counted at the end of the study.^24^

### Biochemical assays

Eight milliliter of venous blood was drawn after a 12 hours fast. The serum samples were separated from the whole blood by centrifugation at 3500 rpm for 10 minutes (Orum Tadjhiz Centrifuge, Iran), at room temperature; serum samples were frozen immediately at -70°C until assay (at the end of the study). The samples were analyzed at the Drug Applied Research Center (Tabriz University of Medical Sciences, Tabriz, Iran). TC was measured by CHOD-PAP kit (Parsazmun kits, Karaj, Iran); cholesterol esterase and cholesterol oxidase method were applied in the assay. To measure TG, GOP-PAP kit (Parsazmun kits, Karaj, Iran) was used; TG was assayed using glycerol phosphate oxidase. CHOD-PP-PAP kit (Parsazmun kits, Karaj, Iran) was used to measure HDL-C, which measured HDL-C after precipitation of the apolipoprotein B-containing lipoproteins. TC, TG and HDL-C concentrations were read by an autoanalyzer (Abbott, model Alcyon 300, Philippines). Friedewald formula was employed to calculate serum LDL-C concentration.^25^

### Statistical analyses

SPSS version 20.0 software (SPSS Inc, Chicago, IL, USA) was used to analyze the experimental data and the results were expressed as mean (SD) for normally distributed quantitative data, median (percentiles 25 and 75) for quantitative data not normally distributed, and frequency (percent) for qualitative data. The normality of data distribution was determined by Kolmogorov-Smirnov test. To compare the 2 intervention groups for the variables at baseline, independent samples *t* test, Mann-Whitney U test, chi square and Fisher exact test were used. Paired samples *t* test, Wilcoxon signed-rank test and Sign test were used to assess within group changes, throughout the study. To compare the 2 groups at the end of the trial, analysis of covariance (ANCOVA) was used, adjusting for the baseline measures, age and menopausal status. Differences with *P *< 0.05 considered statistically significant.

## Results

**Figure 1:**
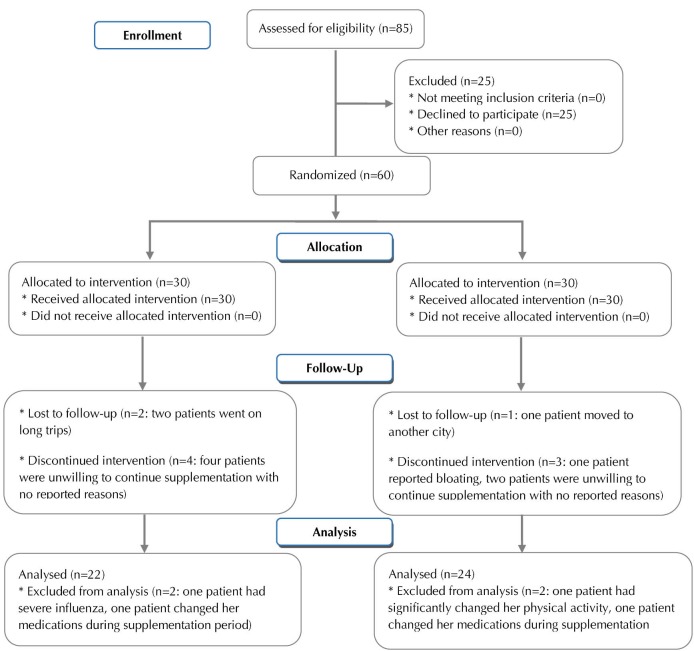
CONSORT flow diagram of the study.

Figure 1 presents the Consolidated Standards of Reporting Trials (CONSORT) flow diagram of the study. The study recruited in September 2012 and ended in November 2012. Ten women withdrew from the trial for reasons irrelevant to the study. Four patients (2 patients in each group) were dropped out of analyses for they had not followed the study protocol; 22 patients in probiotic group and 24 patients in placebo group were analyzed. Compliance with the supplements was good based on capsule counts; no adverse effects were reported. The analyses were performed by original assigned groups. There were no between-group differences for baseline characteristics of the participants; between- and within-group differences were insignificant for anthropometric measures (Table 1). Physical activity and state-trait anxiety levels also remained almost unchanged during the study.^19^ By the end of the study, no significant differences were revealed between the 2 groups for energy and macronutrient intake at baseline; within-group changes were insignificant as well (Table 2).

Table 3 presents the results for serum TC, HDL-C, LDL-C and TG. TC, LDL-C and TG mean values were within normal range, for our patients. No statistically significant differences were observed between the 2 groups for serum lipids at baseline, Within-group changes and percentage of the changes were also insignificant in both groups. At the end of the study, ANCOVA analyses revealed no significant between-group differences for serum lipids.

## Discussion

In the present clinical trial, we found no beneficial effects of the probiotic supplement containing *L. casei* on serum lipids of RA patients.

Anti-inflammatory therapies increase serum lipids in RA subjects,^26,27^ and chronic inflammation impairs the normal cardioprotective function of HDL-C.^28^ Adverse changes in lipid profile following anti-rheumatic therapies, have not been correlated with increased risk of CVD in RA subjects,^29^ but necessitate periodic monitoring of serum lipids particularly when anti-rheumatic therapies are intensified.^28^ Administering statins can normalize serum lipid levels and decrease CVD risk in RA patients in RA patients^30^; however, statins have side effects especially myopathy and rhabdomyolysis^31^; thus, thinking of safer ways for improving lipid profile in RA patients may be helpful. With respect to hypolipidemic effects claimed for some probiotics, we hypothesized that *L. casei* 01 may have a positive influence on serum lipids in RA.

The major mechanisms for hypolipidemic effects of probiotics include reduced cholesterol absorption in the gut, enzymatic deconjugation of bile salts, incorporation of cholesterol into their cell membranes, and conversion of cholesterol to coprostanol.^32,33^ Moreover, short chain fatty acids (SFAs) produced by probiotics inhibit hydroxymethylglutaryl coenzyme A reductase (HMG-CoA reductase) activity and may decrease cholesterol production in the liver.^34^

Unlike animal studies, clinical trials have come up with controversial results regarding the hypolipidemic effects of probiotics. Jahreis et al showed that consuming probiotic sausage containing 5×10^9^ CFU of *L. paracasei* LTH 2579 for 5 weeks had no influence on serum lipids in hypercholesterolemic subjects.^35^ Also, supplementing hypercholesterolemic patients with 6×10^10^ CFU of *L. acidophilus* for 6 weeks did not improve lipid profile.^36^ Receiving probiotic capsules containing 4×10^9^ CFU of *L. fermentum* for 10 weeks,^20^ or supplements with 4×10^10^ CFU of *L. rhamnosus* and *Propionibacterium freudenreichii* for 4 weeks,^37^ did not significantly change serum lipids in hypercholestrolemic participants, either. The results of our study were in accord with these studies.

On the contrary, some clinical trials have reported hypocholesterolemic effects for probiotics. In a study by Ataie-Jafari et al, consuming probiotic yogurt containing 3×10^8^ CFU of *L. acidophilus* and *Bifidobacterium lactis* for 6 weeks reduced TC significantly.^12^ In another trial, TC, LDL-C, and TC/HDL-C and LDL-C/HDL-C ratios decreased in diabetic subjects following six weeks of probiotic yogurt intake, which had 10^9^ CFU of *L. acidophilus* and 9×10^9^ CFU of *B. lactis*.^13^ Probiotic yogurt consisting of a variety of probiotic strains for 8 weeks resulted in a significant reduction of TC and LDL-C in metabolic syndrome patients, as well.^14^ Receiving milk products fermented by *B. longum* BL1 (9×10^9^ CFU/d) for 4 weeks significantly diminished TC particularly in those with mild hypercholesterolemia.^11^

The inconsistent findings on hypocholesterolemic effect of probiotics could be partly justified by the varying strains and doses of probiotics administered, different study durations and health status of the study participants.^33^ To obtain the best anti-inflammatory effects from the intervention in our RA patients,^19^ we used a strain of *L. casei*,^38-42^ and at a lower dosage than the previous trials.^43,44^ Our study duration was similar to studies with significant results and may not have implicated the outcomes of our trial. Serum lipids concentrations were within the normal range at baseline among our participants; it is probable that hypercholesterolemic subjects draw more significant benefits from probiotics. It is also noteworthy that the preferred carrier for probiotics may be dairy foods than capsules, when lowering serum lipids is aimed^36-37,45^; freeze-dried bacteria, when administered in capsules, may not have sufficient time to become metabolically active in the intestine and exert their hypolipidemic effects, before being flushed into colon.^36^ We could not use dairy as delivery vehicles for our probiotics in the present clinical trial. Because, most RA patients avoid consuming dairy foods and believe that these products worsen their pains; this may be explained by the potential antigens in dairy products which trigger immune responses.^46,47^

The major limitation in our study was that the patients did not agree to provide their feces samples for us to confirm colonization of the supplemented probiotic in their gut. However, according to previous in vitro studies,^48-50^ and due to the positive results obtained for anti-inflammatory effects of this strain in our patients,^19^ it is most probable that *L. casei* 01 was sufficiently colonized in the gut; some previous studies have also failed to collect feces samples of the participants, and have relied on only capsule counts.^24^ The strength point of our trial was controlling for dietary intakes, physical activity and anxiety levels during the study; any significant changes in these parameters could have confounded our results. Also, the patients were recruited from 2 different clinics and were of different social class and economic status, and the age range was wide for our participants; thus, it is most probable that our study had external validity.

## Conclusion

Our results found no beneficial effects of *L. casei* 01 supplementation on lipid profile of women suffering from RA. It is suggested that probiotic foods (other than dairy products) and other strains with confirmed hypolipidemic effects be administered in the future studies. Also, conducting the future clinical trials in a subgroup of RA patients with hypercholesterolemia may reveal significant results.

## Ethical approval

The present study was approved by the ethics committee of Tabriz University of Medical Sciences (no. 91233) and performed according to the guidelines laid down in the Helsinki Declaration. All participants gave written informed consent after the nature of the procedures was explained for them. The study was registered in the Iranian Registry of Clinical Trials (IRCT) available at: http://www.irct.ir (ID: IRCT201306264105N14).

## Competing interests

We declare herein that we have no conflict of interest.

## Authors’ contributions

EVM, LVM, MAJ, AHR, KI, BA, and EVM contributed to conception and design of the study, acquisition, analysis and interpretation of data, drafting the article, final approval of the version to publish, accountable for all aspects of the work; LVM, MAJ and AHR contributed to conception and design of the study, acquisition, analysis and interpretation of data, revision of the article, final approval of the version to publish, accountable for all aspects of the work; and KI and BA contributed to conception and design of the study, acquisition, analysis and interpretation of data, revision of the article, final approval of the version to publish, accountable for all aspects of the work.

## Acknowledgements

We sincerely thank all the patients for participating in our study. We also appreciate Dr. Sakineh-Khatoun Sharif for her precious comments on the study protocol and collaboration. The present study was funded by the Research Vice Chancellor of Tabriz University of Medical Sciences, Tabriz, Iran.

**Table 1:** Baseline characteristics of the patients

	**Placebo group (n=24)**	**Probiotic group (n=22)**
Age (y)	44.29 (9.77)	41.14 (12.65)^a^
Height (cm)	156.02 (6.40)	158.16 (6.78)^a^
Weight (kg)	68.56 (11.96)	69.29 (11.47)^a^
BMI (kg/m^2^)	28.08 (4.03)	27.70 (4.16)^a^
SBP (mm Hg)	121.87 (22.54)	119.89 (16.30)^a^
DBP (mm Hg)	74.58 (12.04)	75.91 (9.18)^a^
Duration of RA (y)	4.75 (3.0, 9.0)	5.25 (3.75, 10.0)^b^
Menopausal status (no.)		
Premenopausal	17 (70.8)	15 (68.2)
Postmenopausal	7 (29.2)	7 (31.8)^b^
Current medication (no.)		
Methotrexate	20 (83.3)	15 (68.2)^c^
Hydroxychloroquine	18 (75.0)	18 (81.8)^d^
Prednisolone	23 (95.8)	21 (95.5)^d^

Abbreviations: BMI: body mass index; RA: rheumatoid arthritis; SBP: systolic blood pressure; DBP: diastolic blood pressure. Mean (SD) is reported for age, height, weight, BMI, SBP and DBP. Median (percentiles 25 and 75) is presented for duration of RA. Frequency (percent) is reported for menopausal status and current medication. ^a^ *P* > 0.05 based on independent *t* test. ^b^ *P* > 0.05 based on Mann-Whitney U test. ^c^ *P* > 0.05 based on chi-square test. ^d^ *P* > 0.05 based on Fisher exact test.

**Table 2:** Dietary intake of subjects throughout the study

		**Placebo group (n = 24)**	**Probiotic group (n = 22)**
Energy (Cal)	Baseline	1699.68 (416.49)	1689.82 (358.32)^a^
	End of study	1696.41 (423.30)^b^	1694.82 (329.17)^ab^
Protein (g)	Baseline	51.35 (16.73)	51.86 (15.80)^a^
	End of study	53.00 (14.23)^b^	51.21 (14.17)^ab^
Fat (g)	Baseline	51.06 (17.25)	55.60 (11.12)^a^
	End of study	55.85 (18.13)^b^	59.80 (14.02)^ab^
PUFA (g)	Baseline	10.37 (4.07)	12.36 (3.15)^a^
	End of study	12.37 (5.59)^b^	12.86 (4.87)^ab^
MUFA (g)	Baseline	18.02 (7.05)	19.58 (5.07)^a^
	End of study	20.34 (7.81)^b^	20.41 (5.67)^ab^
SFA (g)	Baseline	11.63 (6.14)	11.15 (5.60)^a^
	End of study	12.52 (5.51)^b^	13.98 (6.46)^ab^
Fiber (g)	Baseline	14.54 (7.82)	11.80 (5.90)^a^
	End of study	11.52 (4.48)^b^	11.85 (4.56)^ab^

Abbreviations: PUFA, polyunsaturated fatty acids; MUFA, monounsaturated fatty acids; SFA, saturated fatty acids. Mean (SD) are presented for the measures. ^a^ *P* > 0.05 based on Independent *t* test. ^b^ *P* > 0.05 based on Paired *t* test.

**Table 3:** Effects of 8 weeks of probiotic supplementation as compared with placebo on serum lipids in female patients with rheumatoid arthritis

	**Placebo ( n= 24)**	**Probiotic (n = 22)**	**Mean difference (95% CI),** ***P*** ** value**
TC (mg/dL)			
Baseline	185.62 (34.92)	176.22 (41.31)	-9.40 (-32.06, 13.27), 0.408^a^
End of study	183.04 (47.72)	174.32 (31.44)	-0.18 (-0.65, 0.29), 0.801^c^
Mean difference (95% CI), *P* value^b^	-2.58 (-13.30, 18.47), 0.740	-1.91 (-7.01, 10.83), 0.661	
HDL-C (mg/dL)			
Baseline	39.33 (9.12)	38.11 (9.92)	-1.22 (-6.88, 4.43), 0.665^a^
End of study	38.00 (7.73)	37.67 (9.52)	-1.66 (-19.28, 15.95), 0.663^c^
Mean difference (95% CI), *P* value^b^	-1.33 (-1.85, 4.52), 0.396	-0.44 (-1.81, 2.68), 0.690	
LDL-C (mg/dL)			
Baseline	124.25 (30.57)	118.04 (33.00)	-6.21 (-25.10, 12.68), 0.511^a^
End of study	124.25 (43.85)	117.05 (23.19)	-2.73 (-19.17, 13.72), 0.666^c^
Mean difference (95% CI), *P* value^b^	0.00 (-14.96, 14.95), 0.999	-0.98 (-7.17, 9.13), 0.805	
TG (mg/dL)			
Baseline	110.08 (36.18)	100.41 (34.47)	-9.67 (-30.72, 11.37), 0.359^a^
End of study	103.92 (34.13)	97.95 (44.72)	0.12 (-19.76, 20.00), 0.900^c^
Mean difference (95% CI), *P* value^b^	-6.17 (-8.74, 21.07), 0.401	-2.45 (-13.13, 18.04), 0.747	

Abbreviations: TC, total cholesterol; HDL-C,high-density lipoprotein-cholesterol; LDL-C: low-density lipoprotein-cholesterol; TG,triglyceride. Mean (SD) are presented for data. ^a^ Independent *t* test. ^b^ Paired *t* test. ^c^ Based on ANCOVA adjusted for baseline measures, age and menopausal status.
